# The influence of gestational age in the psychometric testing of the Bernese Pain Scale for Neonates

**DOI:** 10.1186/s12887-018-1380-8

**Published:** 2019-01-15

**Authors:** Karin Schenk, Liliane Stoffel, Reto Bürgin, Bonnie Stevens, Dirk Bassler, Sven Schulzke, Mathias Nelle, Eva Cignacco

**Affiliations:** 1 Division of Midwifery, Department of Health Professions, Bern University of Applied Sciences, Murtenstrasse 10, 3008 Bern, Switzerland; 2 Neonatalogy, Children’s Hospital, University Hospital of Bern, Bern, Switzerland; 3 Lawrence S. Bloomberg Faculty of Nursing and Faculties of Medicine and Dentistry, University of Toronto, Toronto, ON Canada; 4 Department of Neonatology, University Hospital Zurich and University of Zurich, Zurich, Switzerland; 5 Department of Neonatology, University of Basel Children’s Hospital (UKBB), Basel, Switzerland

**Keywords:** Pain assessment, Neonates, Premature infants, Psychometric testing, Contextual factors, Gestational age, Reliability, Validity

## Abstract

**Background:**

Assessing pain in neonates is challenging because full-term and preterm neonates of different gestational ages (GAs) have widely varied reactions to pain. We validated the Bernese Pain Scale for Neonates (BPSN) by testing its use among a large sample of neonates that represented all GAs.

**Methods:**

In this prospective multisite validation study, we assessed 154 neonates between 24 2/7 and 41 4/7 weeks GA, based on the results of 1–5 capillary heel sticks in their first 14 days of life. From each heel stick, we produced three video sequences: baseline; heel stick; and, recovery. Five blinded nurses rated neonates’ pain responses according to the BPSN. The underlying factor structure of the BPSN, interrater reliability, concurrent validity with the Premature Infant Pain Profile-Revised (PIPP-R), construct validity, sensitivity and specificity, and the relationship between behavioural and physiological indicators were explored. We considered GA and gender as individual contextual factors.

**Results:**

The factor analyses resulted in a model where the following behaviours best fit the data: crying; facial expression; and, posture. Pain scores for these behavioural items increased on average more than 1 point during the heel stick phases compared to the baseline and recovery phases (*p* < 0.001). Among physiological items, heart rate was more sensitive to pain than oxygen saturation. Heart rate averaged 0.646 points higher during the heel stick than the recovery phases (*p* < 0.001). GA increased along with pain scores: for every additional week of gestation, the average increase of behavioural pain score was 0.063 points (*SE* = 0.01, *t* = 5.49); average heart rate increased 0.042 points (*SE* = 0.01, *t* = 6.15). Sensitivity and specificity analyses indicated that the cut-off should increase with GA. Modified BPSN showed good concurrent validity with the PIPP-R (*r* = 0.600–0.758, *p* < 0.001). Correlations between the modified behavioural subscale and the item heart rate were low (*r* = 0.102–0.379).

**Conclusions:**

The modified BPSN that includes facial expression, crying, posture, and heart rate is a reliable and valid tool for assessing acute pain in full-term and preterm neonates, but our results suggest that adding different cut-off points for different GA-groups will improve the BPSN’s clinical usefulness.

**Trial registration:**

The study was retrospectively registered in the database of Clinical Trial gov. Study ID-number: NCT 02749461. Registration date: 12 April 2016.

## Background

Acute painful status in preverbal infants is assessed and interpreted by observing measurable behavioural and physiological indicators. An infant who undergoes an invasive procedure may react to pain that is not caused solely by the painful stimulus [1, 2]. Incorporating individual contextual factors, like gestational age (GA) and gender, into pain assessment tools might make them more accurate [3, 4]. The physiological and behavioural dimensions of pain in neonates are measured by several multidimensional pain assessment tools developed over the last three decades [4–6], but experts agree that behavioural, physiological and cortical measures of pain do not converge to reliably depict and assess the phenomenon of pain in such a vulnerable population [7, 8]. Discrepancies and low-to-moderate associations between behavioural (e.g., facial expression) and physiological (e.g., changes in heart rate) indicators of pain [9–12] have sparked ongoing debate about the appropriate dimensionality of pain scales [7]. Infants may also display nonspecific physiological and behavioural pain indicators during stressful experiences that are not painful, which makes it more challenging to accurately assess pain in neonates [13, 14].

Many pain assessment tools are used in neonatal intensive care unit (NICU) settings. Most add behavioural and physiological indicators to a summary score that is then measured against a cut-off that separates pain from no pain [4]. Rigorous psychometric testing has been applied only to a few [15] (e.g., the Premature Infant Pain Profile [16]). Most were validated for a specific GA in tests that assessed acute pain in full-term and healthy preterm infants with higher GA [4]. However, neurodevelopment and the associated ability to react to painful stimulus varies greatly among early and late preterm infants and full-term neonates: neonates with lower GA express less behavioural pain than more mature neonates [17–22]. In neurologically impaired and very ill neonates, and in neonates on medications (e.g., sedatives), pain may be faintly expressed, or not at all [13, 23].

The Bernese Pain Scale for Neonates (BPSN) is a multidimensional pain assessment tool that includes seven subjective items (sleeping, crying, consolation, skin colour, facial expression, posture, and breathing) and two physiological items (changes in heart rate and oxygen saturation) [24]. The BPSN has been used by clinicians since 2001; 46% of Swiss NICUs rely on this tool to assess pain in neonates [25]. The results of the first validation study in the year 2004 suggested that the BPSN is a valid and reliable scale for assessing acute pain in full-term and preterm neonates with different GAs [24]. However, clinical experts have said the tool is less useful for assessing pain in extremely preterm neonates who, for example, always score very low. This feedback and the increasing scientific evidence which indicates that neonates’ pain reaction is influenced by individual contextual factors [1] have motivated us to re-evaluate the tool with sophisticated psychometric tests to assess its accuracy across all GAs.

This study is the first part of a comprehensive BPSN validation and extension study, designed to develop a modified version of the BPSN that includes relevant individual contextual factors in pain assessment. In this first part, we evaluated the BPSN with psychometric tests. The second part of the study will explore the influence of individual contextual factors (e.g., medication, or number of previous painful experiences) on variability in pain reactions across repeated measurement points.

We used psychometric tests to determine the applicability of the BPSN across neonates who ranged from 24 to 42 weeks of GA. We evaluated interrater reliability, the underlying factor structure of the BPSN, and the internal consistency of the scale. We also assessed concurrent validity with the Premature Infant Pain Profile-Revised (PIPP-R; [26]), construct validity, specificity and sensitivity, and determined the relationship between behavioural and physiological indicators of pain. GA groups and gender were considered as individual contextual factors.

Based on the results of the first validation study of the BPSN [24], we hypothesized that the BPSN is a valid and reliable tool for assessing pain in preterm and full-term neonates. Due to feedback from clinical experts concerning difficulties in pain assessment in extremely preterm neonates and the increasing scientific evidence that indicates neonates’ pain reaction is influenced by individual contextual factors [1], we assumed that we will find a difference in pain reaction depending especially on neonates’ GA. Furthermore, we hypothesized only a low-to-moderate association between behavioural and physiological indicators of pain.

## Methods

### Sample and settings

This was a prospective multisite validation study with repeated measurement design. It was conducted in three university hospital NICUs in Switzerland (Basel, Bern and Zurich). The study was approved by the Ethics Committee Bern, the Ethics Committee northwest/central Switzerland, and the Ethics Committee Zurich. Recruitment and data collection were ongoing, from January 1 to December 31, 2016. Data collection was extended in Bern until January 31, 2017, because we needed to recruit more extremely premature neonates. We included premature neonates born between 24 0/7 and 36 6/7 weeks of gestation, if they were expected to undergo 2–5 routine capillary heel sticks in their first 14 days of life. We included full-term neonates born between 37 0/7 and 42 0/7 weeks of gestation, if they were expected to have at least two routine capillary heel sticks during their first 14 days of life. We needed parental permission to include preterm and full-term neonates. We excluded neonates if they had had a high-grade intraventricular haemorrhage (grades III and IV), if they had a severe life-threatening malformation or suffered from any condition that caused partial or total loss of sensitivity, if they had an arterial cord pH < 7.15 at birth, if they had surgery for any reason, or if they had a congenital malformation that affected brain circulation and/or cardiovascular system.

### Recruitment and data collection procedures

Neonates were recruited by consecutive sampling and then stratified according to GA at birth [27]. Trained study assistants in each study centre identified potentially eligible neonates and informed their parents of the aim and purpose of the study. After parents granted written informed consent, trained study assistants videotaped neonates (using a HC-V757 high-definition camcorder manufactured by Panasonic, Osaka, Japan) during their next 1–5 routine capillary heel sticks. For each heel stick, we produced three video sequences: baseline, heel stick, and recovery phases. Each video sequence began by focusing on the face of the neonate for at least 1 minute to allow adequate assessment of facial activity and cry. Thereafter, the infant’s body was recorded for at least 1 minute. Bedside nurses were asked not to handle the neonates before the baseline phase was recorded, to avoid additional distress that could change the measurement. During the heel stick procedure, the neonates were lying in their incubator (or crib) and the position of the infants was unchanged for the video recording. The baseline phase was recorded 2 to 3 min before the beginning of the heel stick procedure. Afterwards, the bedside nurse warmed the neonate’s heel and gave the infant a dose of 24% oral sucrose (0.2 ml/kg bodyweight) to relieve pain [28]. When the nurse disinfected the neonate’s heel, the recording of the heel stick phase began. First, the neonate’s face was recorded, until the nurse finished the heel stick procedure, which lasted at least a minute. Then the infant’s body was recorded for at least one more minute. The recovery phase began immediately after the heel stick phase was recorded. During each phase of the heel stick procedure, our study assistants recorded the infant’s highest heart rate and lowest oxygen saturation measurement from the infant’s monitors, which tracked this data continuously.

Each video sequence was checked for quality and digitally elaborated by trained study assistants in Final Cut Pro X [29] video editing software. We removed any information that could have revealed the heel stick phase to the raters to ensure continued blindness. The video sequences were uploaded onto a web-based rating tool developed for our study. Uploaded sequences were randomized by sequence number, phase, and presentation order. Five nurses who were working in a NICU and were experienced in using the BPSN (*Mean* = 8.3 years of experience, *SD* = 6.1, *Range* = 3.5–15 years) retrieved the video sequences from the web-based platform and independently rated the behavioural pain expression of the neonates using the BPSN and the PIPP-R. The nurses were trained to use and score the PIPP-R.

### Measures

Pain reaction was measured with the BPSN [24] and the PIPP-R [26]. Each of the nine items of the BPSN is rated on a 4-point Likert scale (0, 1, 2, and 3), and then the scores are summed. On the BPSN total score, which includes seven subjective items (i.e., sleeping, crying, consolation, skin colour, facial expression, posture, and breathing), and two physiological items (i.e., changes in heart rate and oxygen saturation), the scores of 11 or more points indicate pain (BPSN total scores range from 0 to 27). In a first validation study in the year 2004 [24], the BPSN showed good construct validity among neonates with GAs between 27 and 41 weeks (*n* = 12); BPSN scores were significantly higher during painful (*M* = 15.96, *SD* = 5.7) compared to non-painful (*M* = 2.32, *SD* = 1.6, *p* < 0.001) situations. Furthermore, the correlations between the BPSN and the Visual Analog Scale (VAS; *r* = 0.855, *p* < 0.0001) and the PIPP (*r* = 0.907, *p* < 0.0001) were high, as well as the interrater (*r* = 0.86–0.97) and intrarater reliability (*r* = 0.98–0.99) of the BPSN [24]. In our study, five independent blinded raters watched the videos to rate the seven subjective items. Both physiological indicators were captured from the neonate’s monitoring records during video recordings. Because the raw data on heart rate, oxygen saturation and breathing rate in the baseline phase was used to calculate differences during the heel stick and recovery phases, we set the baseline scores of these items to zero, and retrospectively converted the raw data between baseline, heel stick, and recovery phase into BPSN scores that ranged between 0 and 3.

The PIPP-R is a well validated pain assessment tool for use with premature and full-term neonates, widely used in North America in clinics and for research [16, 26, 30, 31]. The PIPP-R includes three behavioural indicators (brow bulge, eye squeeze, and naso-labial furrow) and two physiological indicators (heart rate and oxygen saturation). Each indicator is rated on a 4-point Likert scale (0, 1, 2, and 3). The PIPP-R accounts for GA and baseline behavioural state as contextual factors. Neonates with younger GAs and neonates in quiet sleep state score the highest, but they are only factored in if the infant’s behavioural and physiological sub score is ≥1 [26]. Zero points indicate no pain or perhaps no response to pain, 1–6 points indicate low pain, 7–12 points indicate moderate pain, and ≥ 13 severe pain. Total PIPP-R scores range from 0 to 21 for neonates with GA < 28 weeks in a quiet and sleep baseline behavioural state, and from 0 to 15 for full-term neonates in an active and awake baseline behavioural state [26]. The PIPP-R shows beginning construct validity [30]; PIPP-R scores were significantly higher during painful (*M* = 6.7, *SD* = 3.0) compared to non-painful (*M* = 4.8, *SD* = 2.9; *p* < 0.001) procedures among full-term and preterm neonates with GAs as young as 26 weeks of gestation (*n* = 202). In addition, the PIPP-R showed good interrater reliability between nurses and pain experts (*R*^2^ = 0.87–0.92; *p* < 0.001), and nurses reported that the PIPP-R is a feasible and appropriate pain assessment tool [30]. In our study, both physiological indicators were captured from the neonate’s monitoring records and converted into PIPP-R scale values like the physiological indicators of the BPSN. The behavioural indicators and behavioural state were rated from the videos by the same five independent raters. We calculated interrater reliability of the three behavioural items with a two-way random-effects, absolute agreement, single measure model that ranged from 0.750 to 0.842 (*Mdn* = 0.803) in the heel stick phases of the five measurement points.

We retrieved individual contextual factors retrospectively from patient charts [27] and will publish a separate paper describing their influence on the variability of pain reaction across repeated measurement points.

### Sample size and power

Our target sample size of 150 neonates was based on an a priori power analysis of the hypothesized association between the BPSN and GAs at baseline. That analysis was based on data from a previous study (*n* = 71; [32]) and a descriptive-explorative analysis (*n* = 23); it assumed a Type I error probability of 5%, a power of 80%, and at least three documented baseline heel sticks per study infant.

### Data analysis

Factor analyses explored the structure of the BPSN and measurement invariance. Psychometric tests examined interrater reliability, internal consistency, construct validity, concurrent validity with the PIPP-R [30], association between behavioural and physiological items, and sensitivity and specificity. Because the sample was heterogeneous, we also conducted analyses for different GA-groups. We used the statistics programs SPSS [33] and R [34] for all analyses. Space restriction limit us to reporting mainly our results from the heel stick phases. In this comprehensive validation study, we did multiple testing of outcome data arising from individual neonates. Correction of *p*-values with Bonferroni adjustment [35] would not have rendered findings non-significant. Therefore, all *p*-values are presented uncorrected for multiple testing unless otherwise specified. A *p*-value < 0.05 was considered statistically significant.

## Preliminary analyses

Exploratory analyses described the data and looked for anomalies that could reduce the validity of the data analysis. We used descriptive and frequency statistics to describe sample characteristics and each rater’s pain scores.

## Missing values

We analysed the ratings of the 1′817 video sequences for the volume and pattern of missing data, since single items of the BPSN and the PIPP-R could be rated “non-evaluable”. Because it is impossible to compute BPSN and PIPP-R sum scores when an item was not rated, we used multiple imputation [36] and the R-package *partykit* [37] to derive those scores by replacing the values of non-rated items with random substitutes generated from conditional inference regression trees [38]. We generated five data sets, so there were five variants on the BPSN and PIPP-R sum scores.

## Interrater reliability

Intraclass correlation coefficients (ICCs) and their 95% confidence intervals were calculated to determine interrater reliability of the seven subjective BPSN-items [39, 40]. Since pain reaction of a neonate is rated by a single nurse in the clinical setting, and pain level scores were central to our outcome, we assessed interrater reliability with a two-way random-effects, absolute agreement, single measure model [41]. ICC coefficients were also calculated with a two-way random-effects, absolute agreement, average measure model, to generate more information about the reliability of the mean ratings provided by the five raters [40]. Each phase of the five measurement points was analysed separately, resulting in 120 ICC coefficients (8 rating scores * 3 phases * 5 measurement points) per model.

## Factor analyses

### Measurement construct

Multiple group longitudinal confirmatory factor analysis [42] was used to evaluate the extent to which individual items correlated with the unobservable pain construct, the predictive performance of the construct, and whether factor loadings were invariant across time and raters. The R-package *lavaan* [43] was used for this analysis. Full maximum likelihood estimates were based on the assumption that data were missing at random.

### Model specification

**Fig. 1:**
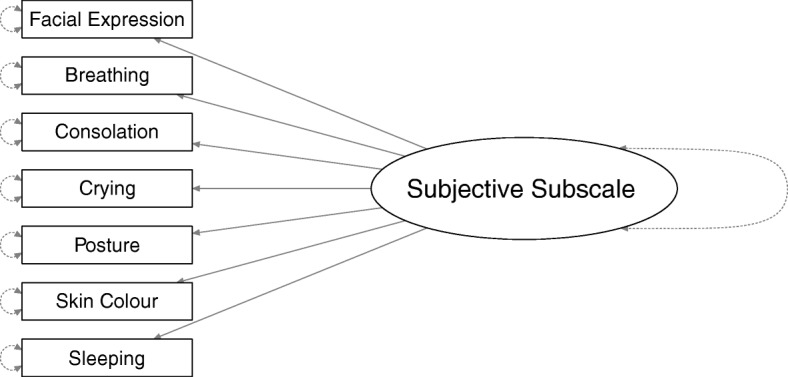
The structure of the factor model used for the subjective subscale of the BPSN

**Fig. 2:**
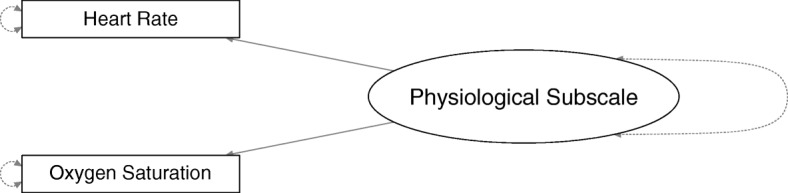
The structure of the factor model used for the physiological subscale of the BPSN

Figures 1 and 2 show the structures of our confirmatory factor analysis (CFA) models for the subjective and physiological subscales. For item selection, we used only data from the heel stick phases of the five measurement points. Measurement invariance tests were based on data from all phases (baseline, heel stick, and recovery) and all measurement points (t1-t5).

**Fig. 3:**
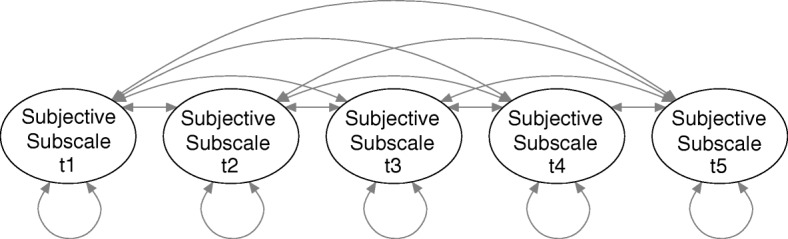
Specified covariances between factors

The longitudinal structure of the data was accounted for by implementing covariances between factors (Fig. 3, structure of the subjective subscale). The covariance structure of factors for the physiological subscale or additional phases or measurement points was implemented as shown.

For the subjective subscale, we stacked the data records of raters, and used the rater as a grouping variable. This specification of this model made it impossible to model covariances between values of the same child measured by different raters. We chose this specification because it did allow us to test invariance of model parameters within and across raters.

### Analytical procedure

We selected items to improve the fit of the CFA model. At estimation, to remove inconsistent items, we restricted loadings of a given item to a common value across raters and measurement points. For both subscales, we estimated several model configurations with at least two items, resulting, for the subjective subscale with 7 items, in 120 models. For the physiological subscale, we used only one model since it included only two items. Selecting the final model was a three-step process. First, we excluded several models with loadings < 0.3 and also excluded models with root mean square errors of approximation (RMSEA) > 0.06, Comparative Fit Indices (CFI; [44]) < 0.95 and Tucker-Lewis Indices < 0.95 (TLI; [45]). The minimal loading size of 0.3 was inspired by Brown [46], and the combinations of cut-offs for the RMSEA, CFI and TLI were inspired by Hu and Bentler [47, 48]. Second, we chose from the remaining models those with the highest number of parameters because we wanted to keep as many appropriate items as possible. Third, we planned to select the model with the highest CFI if Step 2 left us with more than one candidate, but this step turned out to be unnecessary. We found no suitable factor model for the physiological subscale and therefore, we used regression analysis to pick the item most sensitive to pain.

We continued factor analysis by examining measurement invariance across time points within-raters and overall measurement invariance. Only loading (weak) invariance was considered, because other parameters like intercepts and variances could be expected to vary over time and phases. Measurement invariance was examined with Satorra and Bentler’s likelihood ratio test [49] and tests based on the RMSEA, CFI and TLI that used Cheung and Rensvold’s critical values [50].

## Reliability and validity of the modified BPSN

The results of our factor analyses showed that only the behavioural items crying, facial expression, and posture had consistently high factor loadings over time. The physiological items heart rate and oxygen saturation did not load on a common factor and did not correlate with each other. Further analyses showed that the item heart rate was more sensitive to pain than oxygen saturation. We thus decided to exclude the items sleeping, consolation, skin colour, breathing, and oxygen saturation from the BPSN. In following examinations, we used a modified version of the BPSN that included facial expression, crying, and posture, as a behavioural subscale, and heart rate as an additional physiological indicator. Because the results of the measurement invariance analyses showed that the measurement construct measured with the modified behavioural subscale works differently for different raters, we accounted for differences between the raters by either including the raters in the model, or by conducting separate analyses for each rater and then pooling the results.

### Internal consistency and corrected item-total correlation

We evaluated the internal consistency of the modified version of the behavioural subscale that included items facial expression, crying and posture by calculating Cronbach’s *α*. We calculated corrected item-total correlations to analyse correlations between single items and the behavioural subscale. In addition, we calculated the resulting Cronbach’s Alpha when an individual item is removed from the scale (Cronbach’s Alpha if Item Deleted) [51]. Data from each rater were analysed separately, resulting in 75 analyses (5 raters * 3 phases * 5 measurement points), and then we used *cocron* [52], a web interface, to statistically compare the Cronbach’s Alpha coefficients calculated for each rater.

### Correlations between behavioural and physiological indicators of pain

Pearson product-moment correlation coefficients were calculated to establish the association between the modified behavioural subscale of the BPSN and heart rate. Data from each rater were analysed separately, resulting in 50 analyses (5 raters * 2 phases * 5 measurement points). Afterwards, for each phase we examined at each measurement point whether the correlation coefficients calculated for the five raters were statistically different, using the χ^2^-statistics of Steiger [53].

### Construct validity

We compared the level of pain scores between the three phases (baseline, heel stick and recovery) to determine construct validity of the BPSN. We analysed the modified behavioural subscale and heart rate in a linear mixed effect analysis that used the R-package *lme4* [54]. Linear mixed effect analysis allowed us to control variance created by multiple measurement points per subject [55]. The three phases, five measurement points, GA at time of birth, and gender were fixed effects in the model. Neonates and raters were random intercepts. Likelihood Ratio Tests tested the effect of the three phases on the level of pain scores [55].

### Concurrent validity

Pearson product-moment correlation coefficients were calculated to establish concurrent validity between the modified total scores of the BPSN (facial expression, crying, posture, heart rate) and the PIPP-R. Separate analysis were performed for the data of each rater, resulting in 75 analyses (5 raters * 3 phases * 5 measurement points), and afterwards, we examined for each phase at each measurement point if the correlation coefficients calculated for the five raters were not statistically different, again using the χ^2^-test of Steiger [53].

## Specificity and sensitivity analysis

A Receiver-Operating Characteristic (ROC) curve analysis was used to evaluate the ability of the modified BPSN total score to detect pain in neonates and to determine the cut-off value that maximized both sensitivity and specificity [56]. The PIPP-R was the reference value that allowed us to determine sensitivity and specificity; PIPP-R values of ≤6 characterized neonates as experiencing no or low pain; values ≥7 characterized neonates as experiencing moderate to severe pain. We tested whether the area under the curve (AUC) was greater than 0.5 and calculated sensitivity and specificity of the BPSN by using the cut-off values the ROC curve suggested. We performed this analysis separately for the heel stick phases of the five measurement points and the five raters, resulting in 25 ROC curves analysis (5 raters * 5 measurement points), and we averaged the values calculated for each rater.

## Secondary analyses by GA-groups

Infants that ranged from 24 2/7 to 42 5/7 GA at time of birth were included in the primary analyses. Because the sample was heterogenous, we reanalysed the data separately for four GA-groups [57]: extremely preterm neonates (24 0/7–27 6/7 weeks GA); very preterm neonates (28 0/7–31 6/7 weeks GA); moderate to late preterm neonates (32 0/7–36 6/7 weeks GA); and, full-term neonates (37 0/7–42 6/7 weeks GA). Analyses remained the same with exception of the factor and linear mixed model analyses. We could not reanalyse the factor analysis for different GA-groups separately because the sub-samples were too small. In the linear mixed model analyses, GA was already considered as a fixed effect. We did not use Bonferroni adjustment in this subgroup analyses because we exploratively analysed if there were any obvious differences between the four GA-groups.

## Results

### Missing data and sample characteristics

**Fig. 4:**
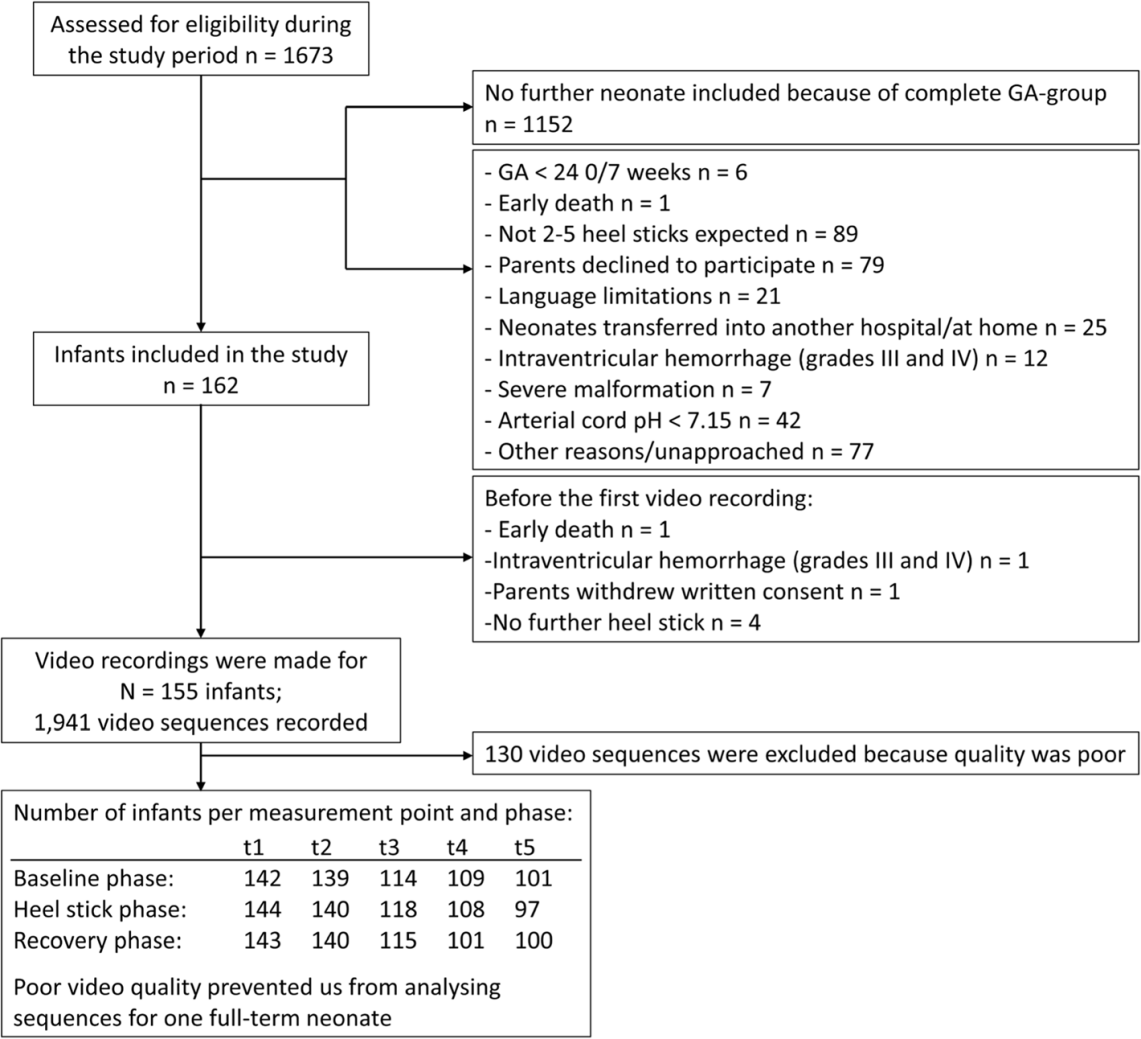
Flow diagram of the recruitment and data collection process

We enrolled a total of 162 neonates in the study; 8 were excluded from data analysis because video sequences were missing or of poor quality. Figure 4 illustrates the flow of recruitment and data collection.

For the five raters, ≤ 1.0% data was missing for the BPSN items sleeping, crying, consolation, skin colour and posture; for facial expression, 0.1 to 4.0% (*Mdn* = 0.8%) data was missing, and for breathing, 0.3 to 8.7% (*Mdn* = 1.9%) was missing. For the PIPP-R, 0.5 to 3.3% (*Mdn* = 1.0%) of data was missing for brow bulge, 0.4 to 3.6% (*Mdn* = 0.7%) for eye squeeze, 0.6 to 28.3% (*Mdn* = 4.3%) for naso-labial furrow, and 0.1 to 0.9% (*Mdn* = 0.4%) for behavioural state. Less than 1% of data was missing for the physiological items heart rate and oxygen saturation.

Mean GA at birth of the total sample was 30.85 (*SD* = 4.5) weeks and ranged from 24.29 to 41.57. Demographic and medical characteristics of the sample are summarized in Table 1.

**Table 1:** Demographic and medical characteristics of the total sample and the four gestational age groups

		Gestational age groups			
	Total Sample	Extremely preterm neonates	Very preterm neonates	Moderate to late preterm neonates	Full-term neonates
Sample, n (%)	154 (100)	50 (32.5)	45 (29.2)	38 (24.7)	21 (13.6)
Sex, n (%)					
- Male	87 (56.5)	31 (62.0)	23 (51.1)	20 (52.6)	13 (61.9)
GA at birth in weeks, mean (SD)	30.85 (4.5)	26.23 (1.2)	29.44 (1.0)	34.21 (1.0)	38. 81 (1.3)
Birth weight in grams, mean (SD)	1630.10 (934.3)	851.40 (196.4)	1285.11 (328.2)	2093.68 (377.5)	3384.52 (811.6)
Number of comorbidities, mean (SD)	5.70 (4.4)	10.06 (4.2)	5.44 (2.4)	2.66 (1.4)	1.38 (1.1)
CRIB score, mean (SD)	3.76 (3.9)	7.50 (3.7)	3.24 (2.8)	1.05 (1.7)	0.86 (1.6)
Way of delivery, n (%)					
- Vaginal-spontan	36 (23.4)	10 (20.0)	4 (8.9)	13 (34.2)	9 (42.9)
- Vaginal-operativ	4 (2.6)	0 (0)	1 (2.2)	2 (5.3)	1 (4.8)
- Planned c-section	23 (14.9)	3 (6.0)	8 (17.8)	7 (18.4)	5 (23.8)
- Emergency c-cection	91 (59.1)	37 (74.0)	32 (71.1)	16 (42.1)	6 (28.6)
Number of birth, mean (SD)					
- Single	104 (67.5)	43 (86.0)	20 (44.4)	21 (55.3)	20 (95.2)
- One of twins	44 (28.6)	4 (8.0)	22 (48.9)	17 (44.7)	1 (4.8)
- One of triplet	6 (3.9)	3 (6.0)	3 (6.7)	0 (0)	0 (0)
Day of life at first measure point, mean (SD)	3.95 (2.0)	4.80 (2.2)	3.56 (1.9)	3.18 (1.0)	4.19 (2.6)

*Note*. *CRIB* Clinical Risk Index for Babies

## Results of descriptive and preliminary analysis

Means of the BPSN total-scale, subjective subscale, and items are summarized in Table 2. Physiological items are not included in this table because they were captured from the neonates’ monitoring records during video recordings and the raw data was retrospectively converted into BPSN scores between 0 and 3. The mean scores for heart rate ranged from 0.47 to 0.76 (*Mdn* = 0.72) during the five heel stick phases, and from 0.03 to 0.11 (*Mdn* = 0.09) during the five recovery phases. The mean scores for oxygen saturation ranged from 0.77 to 1.25 (*Mdn* = 0.86) during the five heel stick phases, and from 0.51 to 0.71 (*Mdn* = 0.61) during the five recovery phases.

**Table 2:** Means of the Bernese Pain Scale for Neonates total-scale and the subjective subscale and items

		Rater A	Rater B	Rater C	Rater D	Rater E
	Phase	Means t1-t5	Means t1-t5	Means t1-t5	Means t1-t5	Means t1-t5
		Range (Median)	Range (Median)	Range (Median)	Range (Median)	Range (Median)
BPSN total-scale	Baseline	0.89–1.14 (1.06)	1.99–2.47 (2.21)	1.31–1.51 (1.38)	4.44–5.15 (4.98)	4.66–4.97 (4.80)
*N* = 81–142	Heel Stick	4.03–4.77 (4.14)	5.98–6.98 (6.33)	4.57–5.41 (4.87)	8.15–9.53 (8.29)	8.00–9.07 (8.52)
	Recovery	1.84–2.30 (2.19)	3.08–3.40 (3.22)	2.37–2.67 (2.46)	5.27–6.27 (6.06)	5.37–5.99 (5.65)
Subjective subscale	Baseline	0.89–1.14 (1.06)	1.99–2.47 (2.21)	1.31–1.51 (1.38)	4.44–5.15 (4.98)	4.66–4.97 (4.80)
*N* = 82–142	Heel Stick	2.51–2.82 (2.68)	4.64–4.96 (4.73)	3.00–3.35 (3.31)	6.59–7.47 (6.84)	6.65–7.04 (6.90)
	Recovery	1.17–1.63 (1.45)	2.39–2.76 (2.51)	1.70–1.97 (1.77)	4.59–5.60 (5.28)	4.66–5.26 (4.94)
Sleeping	Baseline	0.23–0.28 (0.23)	0.39–0.43 (0.41)	0.42–0.51 (0.47)	1.04–1.28 (1.19)	0.89–1.10 (1.05)
*N* = 95–143	Heel Stick	0.39–0.45 (0.42)	0.75–0.91 (0.89)	0.55–0.63 (0.60)	1.19–1.29 (1.23)	1.35–1.46 (1.41)
	Recovery	0.20–0.32 (0.30)	0.40–0.49 (0.41)	0.41–0.51 (0.42)	1.02–1.31 (1.19)	0.89–1.08 (1.06)
Crying	Baseline	0.02–0.06 (0.06)	0.04–0.09 (0.07)	0.04–0.10 (0.06)	0.06–0.11 (0.09)	0.07–0.12 (0.09)
*N* = 96–143	Heel Stick	0.21–0.30 (0.24)	0.30–0.43 (0.36)	0.31–0.42 (0.37)	0.35–0.47 (0.42)	0.36–0.48 (0.43)
	Recovery	0.02–0.06 (0.03)	0.03–0.10 (0.06)	0.03–0.11 (0.07)	0.05–0.11 (0.06)	0.04–0.12 (0.09)
Consolation	Baseline	0.02–0.06 (0.05)	0.05–0.10 (0.09)	0.04–0.12 (0.07)	0.77–1.07 (0.97)	0.03–0.12 (0.08)
*N* = 96–143	Heel Stick	0.21–0.32 (0.21)	0.31–0.48 (0.43)	0.28–0.43 (0.33)	1.19–1.48 (1.26)	0.35–0.55 (0.46)
	Recovery	0.00–0.07 (0.02)	0.03–0.13 (0.06)	0.01–0.15 (0.09)	0.68–0.99 (0.85)	0.02–0.14 (0.11)
Skin colour	Baseline	0.02–0.06 (0.04)	1.00–1.27 (1.11)	0.02–0.06 (0.03)	0.86–1.06 (0.97)	1.51–1.67 (1.61)
*N* = 96–143	Heel Stick	0.05–0.08 (0.07)	1.19–1.29 (1.26)	0.03–0.05 (0.03)	0.99–1.36 (1.07)	1.55–1.79 (1.69)
	Recovery	0.00–0.06 (0.04)	1.05–1.18 (1.13)	0.02–0.04 (0.03)	0.89–1.09 (1.04)	1.48–1.69 (1.53)
Facial expression	Baseline	0.16–0.29 (0.24)	0.17–0.29 (0.19)	0.22–0.32 (0.25)	0.73–0.86 (0.75)	0.83–0.89 (0.87)
*N* = 95–143	Heel Stick	0.50–0.64 (0.61)	0.61–0.69 (0.64)	0.60–0.65 (0.63)	1.01–1.13 (1.06)	1.08–1.18 (1.12)
	Recovery	0.19–0.33 (0.24)	0.09–0.19 (0.17)	1.16–0.26 (0.23)	0.62–0.79 (0.69)	0.80–0.89 (0.87)
Posture	Baseline	0.33–0.49 (0.40)	0.27–0.36 (0.30)	0.45–0.49 (0.48)	0.93–1.04 (0.99)	1.15–1.29 (1.19)
*N* = 97–143	Heel Stick	0.55–0.67 (0.60)	0.69–0.80 (0.78)	0.57–0.71 (0.70)	1.17–1.24 (1.20)	1.38–1.45 (1.41)
	Recovery	0.32–0.43 (0.34)	0.20–0.34 (0.32)	0.37–0.46 (0.41)	0.80–0.94 (0.87)	1.06–1.20 (1.19)
Breathing	Heel Stick	0.47–0.57 (0.50)	0.32–0.65 (0.54)	0.61–0.72 (0.65)	0.50–0.69 (0.64)	0.39–0.62 (0.47)
*N* = 84–142	Recovery	0.35–0.54 (0.45)	0.31–0.46 (0.40)	0.40–0.63 (0.58)	0.49–0.64 (0.53)	0.31–0.58 (0.41)
Raw Scores	Baseline	26.7–27.9 (27.6)	25.7–26.9 (25.8)	27.8–29.5 (28.1)	26.0–26.9 (26.6)	28.4–30.1 (29.5)
Breathing	Heel Stick	28.6–29.2 (28.5)	26.1–27.8 (27.0)	28.2–29.9 (28.9)	27.2–28.3 (27.5)	29.4–30.4 (30.0)
*N* = 91–142	Recovery	27.0–28.7 (27.7)	25.3–27.1 (26.2)	27.4–29.3 (28.3)	26.4–27.4 (26.6)	28.9–30.1 (29.7)

*Note*. N = number of neonates included in the analysis. This number varies because of differences in the amount of missing data between the raters at each measurement point and differences in the number of neonates included at each point of measurement

## Interrater reliability

We derived the results of our interrater reliability analyses by calculating two-way random-effects, absolute agreement models. The results are summarized in Table 3. We again excluded heart rate and oxygen saturation. Interrater agreement for the items crying, consolation, facial expression, and posture tended to decrease across the five measurement points.

**Table 3:** Intraclass Correlation Coefficients and their 95% confident intervals for the single items of the Bernese Pain Scale for Neonates

	Heel Stick Phase 1	Heel Stick Phase 2	Heel Stick Phase 3	Heel Stick Phase 4	Heel Stick Phase 5
	ICC [95%CI]	ICC [95%CI]	ICC [95%CI]	ICC [95%CI]	ICC [95%CI]
Sleeping					
N	135	139	117	105	93
Single measures	0.215 [0.13–0.31]	0.267 [0.18–0.36]	0.211 [0.13–0.30]	0.185 [0.11–0.28]	0.221 [0.13–0.33]
Average measures	0.578 [0.43–0.69]	0.646 [0.52–0.74]	0.572 [0.43–0.69]	0.532 [0.37–0.66]	0.586 [0.43–0.71]
Crying					
N	138	140	117	107	94
Single measures	0.773 [0.72–0.82]	0.694 [0.63–0.76]	0.721 [0.65–0.78]	0.719 [0.65–0.78]	0.655 [0.57–0.73]
Average measures	0.945 [0.93–0.96]	0.919 [0.89–0.94]	0.928 [0.90–0.95]	0.927 [0.90–0.95]	0.905 [0.87–0.93]
Consolation					
N	140	140	117	108	94
Single measures	0.453 [0.31–0.58]	0.381 [0.22–0.53]	0.420 [0.27–0.55]	0.319 [0.16–0.48]	0.257 [0.11–0.41]
Average measures	0.805 [0.69–0.87]	0.755 [0.58–0.85]	0.784 [0.65–0.86]	0.701 [0.48–0.82]	0.634 [0.38–0.78]
Skin colour					
N	141	138	115	108	96
Single measures	0.074 [0.02–0.14]	0.049 [0.03–0.37]	0.073 [0.02–0.15]	0.045 [0.00–0.10]	0.072 [0.01–0.15]
Average measures	0.285 [0.09–0.45]	0.205 [0.03–0.37]	0.284 [0.08–0.46]	0.189 [0.01–0.36]	0.280 [0.06–0.47]
Facial expression					
N	135	130	112	102	92
Single measures	0.655 [0.53–0.75]	0.555 [0.43–0.66]	0.558 [0.45–0.66]	0.500 [0.37–0.62]	0.514 [0.37–0.64]
Average measures	0.905 [0.85–0.94]	0.862 [0.79–0.91]	0.863 [0.80–0.91]	0.833 [0.75–0.89]	0.841 [0.74–0.90]
Posture					
N	141	139	117	108	97
Single measures	0.551 [0.38–0.68]	0.487 [0.31–0.63]	0.536 [0.38–0.66]	0.400 [0.25–0.54]	0.342 [0.21–0.48]
Average measures	0.860 [0.75–0.92]	0.826 [0.69–0.89]	0.852 [0.75–0.91]	0.769 [0.62–0.85]	0.722 [0.57–0.82]
Breathing					
N	119	111	100	95	82
Single measures	0.252 [0.17–0.34]	0.348 [0.26–0.44]	0.334 [0.24–0.44]	0.348 [0.25–0.45]	0.402 [0.30–0.51]
Average measures	0.627 [0.51–0.72]	0.727 [0.64–0.80]	0.715 [0.62–0.79]	0.727 [0.63–0.81]	0.770 [0.68–0.84]
Raw Scores Breathing					
N	128	123	107	106	91
Single measures	0.636 [0.56–0.71]	0.632 [0.56–0.71]	0.674 [0.59–0.75]	0.610 [0.53–0.69]	0.630 [0.54–0.71]
Average measures	0.897 [0.87–0.92]	0.896 [0.86–0.92]	0.912 [0.88–0.94]	0.887 [0.85–0.92]	0.895 [0.86–0.93]

*Note*. ICC = Intraclass Correlation Coefficients, calculated with two-way random-effects, absolute agreement models; [95% CI] = 95% confident intervals of the ICCs

## Factor analyses

### Item selection

**Fig. 5:**
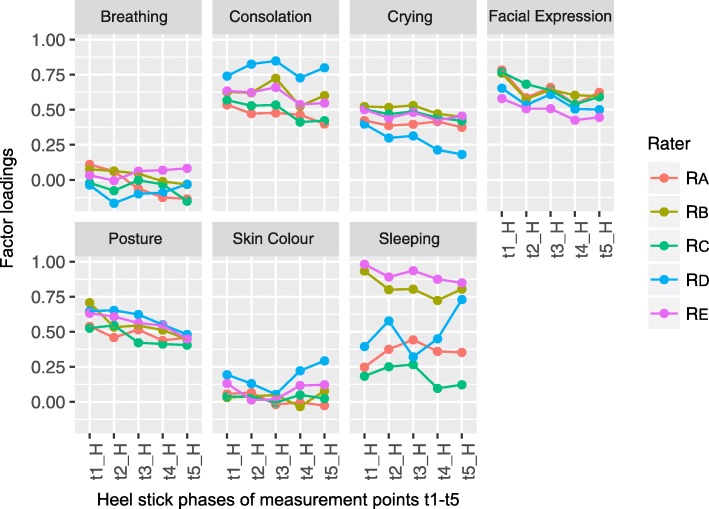
Factor loadings of the baseline factor models for the subjective subscale

**Fig. 6:**
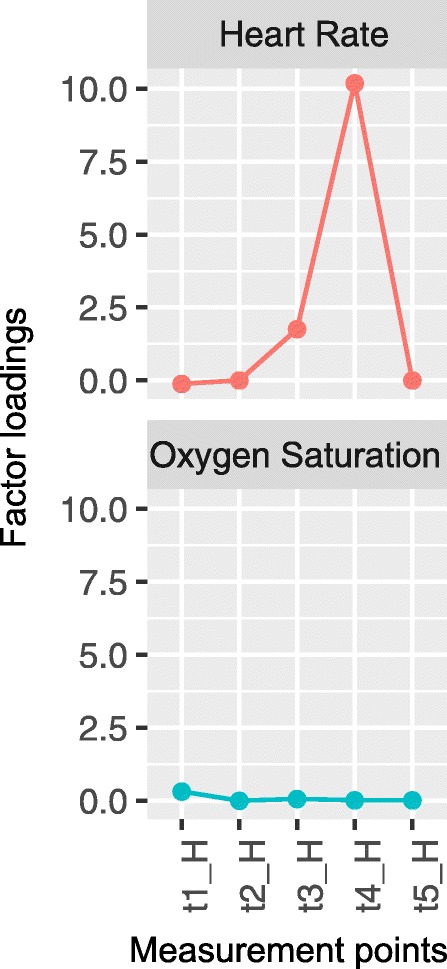
Factor loadings of the baseline factor models for the physiological subscale

First, we used all items and heel stick phases of the five measurement points to estimate the multiple group confirmatory factor models for the subjective and physiological subscale. No parameter restrictions were applied, so that loadings could vary across measurement points and raters. To compare the loadings of all items, we restricted factor variance to 1. Figure 5 shows the estimated factor loadings of the model for the subjective subscale and Fig. 6 for the physiological subscale. For the subjective subscale, loadings for breathing (range = − 0.167-0.110) and skin colour (range = − 0.034-0.293) are low, while loadings for sleeping vary widely between raters (range = 0.096–0.982). Loadings of the remaining items, consolation, crying, facial expression, and posture, seem consistent, but they tend to decrease over time. Rater D’s loadings often conflict with other raters and vary over time.

For the physiological subscale, two loadings exceed by far a value of 1, indicating poor fit between model and data. Additional analyses showed no association between heart rate and oxygen saturation. Pearson product-moment correlations between heart rate and oxygen saturation ranged from *r* = − 0.028 to 0.106 (*Mdn* = 0.017; *p* > 0.05) during the heel stick phases of the five measurement points. Large loadings are probably numerical artefacts and should not be over-interpreted. Because the physiological items did not load on a common factor or correlate with each other, we discarded all but one of the physiological items based on their sensitivity to pain. We analysed the sensitivity to pain of heart rate and oxygen saturation by calculating linear mixed effect models (see next section).

We selected items of the subjective subscale by estimating several configural models with at least two items. In contrast to the model presented in Fig. 5, we restricted factor loadings of a given item to a common value across time points and raters. We excluded models with factor loadings < 0.3, a RMSEA > 0.06 and CFI and TLI < 0.95. This left us with four models, from which we selected the model with the highest number of items. Our final model included only the items crying, facial expression and posture. Table 4 compares model fit indices of the baseline model with all items to the final model with only crying, facial expression, and posture. This improves the CFI and the TLI indices from about 0.8 to 0.95.

**Table 4:** Fit indices of the Baseline and Final Models differ by item inclusions

Model	df	χ^2^	AIC	RMSEA	CFI	TLI	SRMR
Baseline (7 subjective items)	2918	4985	36,875	0.068	0.807	0.803	0.135
Final (Crying, Facial expression, Posture)	472	648	13,575	0.049	0.961	0.957	0.111

*Note*. Model indices: df = degrees of freedom; AIC = Akaike Information Criterion; RMSEA = root mean squared error of approximation; CFI = Bentler’s Comparative Fit Index; TLI = Tucker-Lewis Indices; SRMR = standardized root mean square residual

### Physiological items’ sensitivity to pain

Because the factor analysis indicated that the physiological items heart rate and oxygen saturation do not fit the data well, we next examined these items for their sensitivity to pain. We calculated linear mixed models that included the variables phases, measurement points, GA at time of birth, and gender as fixed effects, and neonates as random intercept. We used Likelihood Ratio Tests to compare a model without the heel stick and recovery phases to a model that included the phases. There was a significant effect of phase on heart rate (χ^2^(5) = 172.91, *p* < 0.001). Heart rate scores during the recovery phases were, on average, 0.646 point lower than scores during the heel stick phases (*SE* = 0.09, *t*-value = − 7.383). Phase also significantly affected oxygen saturation (χ^2^(5) = 33.658, *p* < 0.001). Oxygen saturation scores were, on average, 0.258 points lower during the recovery phases than during the heel stick phases (*SE* = 0.12, *t*-value = − 2.136). We thus decided to use only heart rate for the physiological subscale.

### Measurement invariance

Measurement invariance was examined only for the subjective subscale, since the physiological subscale contained one item. In this analysis, we re-estimated the final model that included crying, facial expression and posture. We used different parameter restrictions: (Free) = all parameters are free; (WRLInv) = within-rater loadings invariance was assumed by restricting loadings of items across time but not across raters; (OLInv) = overall loadings invariance was assumed by restricting loadings across time and across raters. We already applied the OLInv assumption to select items. We next asked if the restricted models fit the data as well as the unrestricted models, and whether factor loadings are (partially) invariant. We performed the same analysis but used only data from the heel stick phase of the five measurement points. Then we used data from all phases and measurement points. Table 5 shows differences between fit indices of the unrestricted and restricted models, including the likelihood ratio test. At a 5% significance level, the zero hypothesis of equal fit or loadings invariance is not rejected for within-rater invariance when we used only data from the heel stick phases, but it was otherwise rejected, most sharply for overall loading invariance (OLInv).

**Table 5:** Difference statistics for measurement invariance testing

Model	Restriction	df	∆ RMSEA	∆ CFI	∆ TLI	∆χ^2^	∆ df	*p* (∆χ^2^)
Heel stick phases of measurement points t1-t5	WRLInv	440	0	0.000	0.002	39	40	0.531
	OLInv	448	0	− 0.015	− 0.015	115	48	0.000
All phases and measurement points	WRLInv	4340	0	−0.007	− 0.006	123	40	0.000
	OLInv	4348	0	−0.024	− 0.025	343	48	0.000

*Note*. WRLInv = within-rater loadings invariance; OLInv = overall loadings invariance; df = degrees of freedom; RMSEA = root mean squared error of approximation; CFI = Bentler’s Comparative Fit Index; TLI = Tucker-Lewis Indices; **∆**χ^2^ = Satorra-Bentler 2010 χ^2^-test statistic

Differences between the fit indices RMSEA, CFI and TLI yield different test results. Using the 1% level rejection areas [50] for the RMSEA, measurement invariance is rejected when the difference is > 0.013, for the CFI, it is rejected when it is < − 0.0085, and, for the TLI, when it is < − 0.0078. Accordingly, within-rater loadings invariance (WRLInv) is never rejected, but overall measurement invariance (OLInv) is always rejected with CFI and TLI, and never with RMSEA.

The tests strongly suggest that the pain measurement construct under consideration works differently for different raters. For within-rater invariance, invariance is not rejected during the heel stick phases; for all data, it is rejected by the *χ*^2^-test but not by RMSEA, CFI and TLI. We may assume approximate invariance, while keeping in mind the results.

## Reliability and validity of the modified BPSN

Our factor analysis and analysis of the physiological items’ sensitivity to pain led us to adopt a modified version of the BPSN for our next analyses. The modified BPSN includes a behavioural subscale (facial expression, crying, and posture) and adds heart rate as a pain indicator.

### Cronbach’s alpha and corrected item-Total correlation

Cronbach’s Alpha, corrected item-total correlation coefficients and the resulting Alpha when an individual item is removed from the scale (Alpha if Item Deleted) for the modified behavioural subscale are summarized in Table 6. During the heel stick phases of the five measurement points, Cronbach’s Alpha coefficients of the five raters differed significantly (*p* < 0.01). Internal consistency of the behavioural subscale tended to decrease over time.

**Table 6:** Cronbach’s Alpha, Corrected Item-Total Correlation and Alpha if Item Deleted calculated for the modified behavioural subscale of the Bernese Pain Scale for Neonates

	Heel Stick Phase 1		Heel Stick Phase 2		Heel Stick Phase 3		Heel Stick Phase 4		Heel Stick Phase 5	
	Median (Range)		Median (Range)		Median (Range)		Median (Range)		Median (Range)	
Cronbach’s α	0.876 (0.841–0.922)		0.848 (0.778–0.885)		0.845 (0.762–0.893)		0.815 (0.725–0.884)		0.825 (0.669–0.852)	
	*r*_*cor*_∗	α**	*r*_*cor*_∗	α**	*r*_*cor*_∗	α**	*r*_*cor*_∗	α**	*r*_*cor*_∗	α**
Crying	0.772	0.850	0.693	0.831	0.689	0.822	0.678	0.768	0.957	0.783
	(0.680–0.875)	(0.801–0.916)	(0.572–0.752)	(0.711–0.881)	(0.559–0.777)	(0.705–0.881)	(0.427–0.772)	(0.689–0.880)	(0.297–0.698)	(0.761–0.846)
Facial expression	0.817	0.773	0.774	0.736	0.781	0.703	0.668	0.696	0.778	0.653
	(0.793–0.907)	(0.700–0.837)	(0.704–0.854)	(0.598–0.783)	(0.699–0.867)	(0.550–0.781)	(0.656–0.845)	(0.444–0.778)	(0.663–0.851)	(0.296–0.682)
Posture	0.741	0.829	0.737	0.790	0.683	0.809	0.694	0.753	0.647	0.781
	(0.679–0.849)	(0.786–0.894)	(0.569–0.794)	(0.718–0.846)	(0.547–0.776)	(0.695–0.866)	(0.567–0.793)	(0.617–0.822)	(0.521–0.687)	(0.519–0.840)

*Note*. Median = Median of the coefficients calculated for each rater separately; Range = Range of the five coefficients calculated for each rater; *r_*cor*_ = Corrected Item-Total Correlation; **α = Cronbach’s Alpha if Item deleted; Number of observations per measurement point *N* = 94–143

### Correlations between behavioural and physiological indicators of pain

We examined the associations between behavioural and physiological indicators of pain with the modified behavioural subscale of the BPSN including the items crying, facial expression, and posture, and the physiological item heart rate. See Table 7 for the correlation coefficients of these analyses. At measurement point 3, the correlation coefficients differed significantly between the five raters (*p* = 0.008), while the correlation coefficients were approximately the same during the other measurement points (*p* > 0.05). When we considered a Bonferroni adjusted *p*-value (*p* < 0.05/10), none of the correlation coefficients would differ significantly between the five raters.

**Table 7:** Pearson product-moment correlation coefficients of the correlations between the modified behavioural Bernese Pain Scale for Neonates-subscale and heart rate

	Heel Stick Phase 1	Heel Stick Phase 2	Heel Stick Phase 3	Heel Stick Phase 4	Heel Stick Phase 5
N	144	140	118	109	97
Median (Range)	0.316* (0.237–0.329*)	0.235 (0.183–0.285)	0.234 (0.102–0.327*)	0.188 (0.155–0.251)	0.305 (0.223–0.379*)

*Note*. Median = Median of the Pearson product-moment correlation calculated for each rater separately; * Bonferroni adjusted *p*-value < 0.001

### Construct validity

To determine construct validity of the BPSN, we compared levels of pain scores of the modified behavioural subscale between the three phases. The residual variance of this analysis was *σ*^2^ = 1.708 (*SD* = 1.307); variances of the random effects were *σ*^2^ = 0.354 (*SD* = 0.595) for neonates and *σ*^2^ = 0.391 (*SD* = 0.625) for raters. Phases significantly affected the level of behavioural pain scores (χ^2^(10) = 864.18, *p* < 0.001). Behavioural pain scores in the heel stick phases averaged 1.04 higher than pain scores in the baseline phases, and 1.13 higher than pain scores in the recovery phases. More results are summarized in Table 8. The same analysis was performed for the item heart rate (Table 8). The residual variance of this analysis was *σ*^2^ = 0.588 (*SD* = 0.767) and variance of the random effect neonates was *σ*^2^ = 0.037 (*SD* = 0.191). GA at time of birth significantly affected behavioural pain scores (*SE* = 0.01, *t* = 5.488) and heart rate (*SE* = 0.01, *t* = 6.145). Gender had no effect on behavioural pain scores (*SE* = 0.10, *t* = − 0.170) or on heart rate (*SE* = 0.05, *t* = 0.051).

**Table 8:** Results of the linear mixed modelling analysis for the modified behavioural Bernese Pain Scale for Neonates-subscale and heart rate

	**Behavioural Subscale**		
**Likelihood Ratio Test**	**χ** ^**2**^	**df**	***p*** **-value**
**Phases**	864.18	10	< 0.001
**Fixed effects**	**Estimated coefficients**	**Std. Error**	***t*** **-value**
Intercept	0.265	0.458	0.579
Baseline phase	−1.041	0.069	−15.008
Recovery phase	−1.134	0.069	−16.040
Measurement point 2	− 0.130	0.070	−1.852
Measurement point 3	0.079	0.074	1.077
Measurement point 4	− 0.078	0.076	−1.038
Measurement point 5	0.097	0.079	1.238
GA at time of birth	0.063	0.012	5.488
Gender (female)	−0.017	0.101	−0.170
Measurement point 2 * Baseline	0.251	0.099	2.538
Measurement point 2 * Recovery	0.309	0.098	3.140
Measurement point 3 * Baseline	0.147	0.104	1.419
Measurement point 3 * Recovery	0.071	0.103	0.682
Measurement point 4 * Baseline	0.308	0.106	2.919
Measurement point 4 * Recovery	0.354	0.105	3.359
Measurement point 5 * Baseline	0.062	0.109	0.569
Measurement point 5 * Recovery	0.067	0.109	0.614
	**Item Heart rate**		
**Likelihood Ratio Test**	**χ** ^**2**^	**df**	***p*** **-value**
**Phases**	172.91	5	< 0.001
**Fixed effects**	**Estimated coefficients**	**Std. Error**	***t*** **-values**
Intercept	−0.563	0.221	−2.547
Recovery phase	−0.646	0.088	−7.383
Measurement point 2	0.023	0.089	0.260
Measurement point 3	−0.199	0.093	−2.139
Measurement point 4	−0.141	0.095	−1.477
Measurement point 5	0.117	0.099	1.183
GA at time of birth	0.042	0.007	6.145
Gender (female)	0.003	0.054	0.051
Measurement point 2 * Recovery	0.021	0.126	0.167
Measurement point 3 * Recovery	0.206	0.131	1.578
Measurement point 4 * Recovery	0.155	0.133	1.160
Measurement point 5 * Recovery	−0.032	0.138	−0.231

*Note*. χ^2^ = Chi-square value; df = degrees of freedom; *N* = 154. Bonferroni adjusted *p*-value < 0.025

### Concurrent validity

We examined the concurrent validity between the modified total score of the BPSN and the PIPP-R. See Table 9 for the correlation coefficients of these analyses. The correlation coefficients of the five raters were the same in about half of the cases. They differed significantly at measurement point 1 (*p* = 0.010) and measurement point 4 (*p* = 0.045). With a Bonferroni adjusted *p*-value (*p* < 0.05/15), none of the correlation coefficients differed significantly between the five raters.

**Table 9:** Pearson product-moment correlation coefficients of the correlations between the total scores of the modified Bernese Pain Scale for Neonates and the PIPP-R

	Heel Stick Phase 1	Heel Stick Phase 2	Heel Stick Phase 3	Heel Stick Phase 4	Heel Stick Phase 5
N	144	140	118	109	97
Median (Range)	0.697** (0.652**-0.758**)	0.709** (0.662**-0.735**)	0.688** (0.649-**0.723**)	0.666** (0.636**-0.735**)	0.648** (0.600**-0.711**)

*Note*. Correlation coefficients were calculated for the heel stick phases of the five measurement points (t1-t5); Median = Median of the Pearson product-moment correlation coefficients that were calculated separately for each rater; **p < 0.01; * *p* < 0.05

## Sensitivity and specificity

The results of the ROC analyses to examine sensitivity and specificity of the modified BPSN total score (including crying, facial expression, posture, and heart rate) are shown in Table 10. During the heel stick phases of the five measurement points, a cut-off of 1.5 points fits best to reach a sensitivity of approximately 80% and a specificity of similar accuracy.

**Table 10:** Results of the ROC analyses for the modified Bernese Pain Scale for Neonates total score

		Cut-off points			AUC
Heel stick phase	N	0.5	1.5	2.5	[95% CI]
**t1**	144				
Sensitivity		0.926	**0.853**	0.724	0.863
Specificity		0.515	**0.662**	0.857	[0.800–0.926]
**t2**	140				
Sensitivity		0.908	**0.811**	0.667	0.825
Specificity		0.442	**0.597**	0.805	[0.756–0.894]
**t3**	118				
Sensitivity		0.874	**0.769**	0.631	0.812
Specificity		0.424	**0.672**	0.858	[0.734–0.890]
**t4**	109				
Sensitivity		0.870	**0.750**	0.574	0.812
Specificity		0.484	**0.685**	0.876	[0.730–0.894]
**t5**	97				
Sensitivity		0.869	**0.794**	0.646	0.812
Specificity		0.383	**0.670**	0.879	[0.722–0.902]

*Note*. The PIPP-R was the reference value, with a cut-off point of 6.5 that discriminated between no/low pain (≤ 6 points) and moderate to high pain (≥ 7 points); AUC = Area under the curve; [95% CI] = 95% confidence intervals of the AUC; the results were originally computed separately for each rater and aggregated assuming normal distribution of the parameters; bold-set font = cut-offs with sensitivity and specificity nearest 80%

## Results of the psychometric testing of the BPSN separated by GA-groups

### Interrater reliability

ICCs coefficients of the four different GA-groups are summarized in Table 11. Interrater reliability of the items facial expression, posture and consolation tended to improve as GA increases.

**Table 11:** Intraclass Correlation Coefficients for the subjective Bernese Pain Scale for Neonates-items calculated with two-way random-effects, absolute agreement models

	Extremely Preterm Neonates	Very Preterm Neonates	Moderate to Late Preterm Neonates	Full-term Neonates
	Heel Stick Phases t1-t5 Range (Median)	Heel Stick Phases t1-t5 Range (Median)	Heel Stick Phases t1-t5 Range (Median)	Heel Stick Phases t1-t5 Range
Sleeping				
N	41–47	32–44	20–34	14–20
Single measures	0.175–0.310 (0.260)	0.145–0.356 (0.198)	0.090–0.289 (0.160)	0.155–0.225
Average measures	0.515–0.692 (0.637)	0.459–0.734 (0.553)	0.330–0.670 (0.487)	0.478–0.592
Crying				
N	40–47	33–44	21–35	14–20
Single measures	0.622–0.794 (0.701)	0.538–0.786 (0.716)	0.564–0.783 (0.702)	0.619–0.680
Average measures	0.892–0.951 (0.921)	0.854–0.948 (0.926)	0.866–0.948 (0.922)	0.890–0.914
Consolation				
N	40–47	33–44	21–35	14–20
Single measures	0.227–0.281 (0.257)	0.216–0.565 (0.390)	0.374–0.598 (0.469)	0.389–0.684
Average measures	0.595–0.661 (0.634)	0.579–0.866 (0.761)	0.749–0.881 (0.815)	0.761–0.915
Skin colour				
N	41–48	34–44	21–36	13–19
Single measures	0.010–0.058 (0.051)	0.002–0.104 (0.062)	0.057–0.166 (0.069)	0.071–0.080
Average measures	0.049–0.236 (0.211)	0.011–0.367 (0.248)	0.230–0.498 (0.271)	0.276–0.302
Facial expression				
N	41–46	31–40	20–34	13–19
Single measures	0.392–0.514 (0.436)	0.498–0.698 (0.526)	0.438–0.748 (0.601)	0.616–0.817
Average measures	0.763–0.841 (0.794)	0.832–0.921 (0.847)	0.796–0.937 (0.883)	0.889–0.957
Posture				
N	42–48	34–44	21–35	14–20
Single measures	0.333–0.479 (0.420)	0.369–0.501 (0.472)	0.286–0.685 (0.519)	0.576–0.795
Average measures	0.714–0.821 (0.783)	0.745–0.834 (0.817)	0.667–0.916 (0.839)	0.872–0.951
Breathing				
N	36–41	29–37	17–35	9–14
Single measures	0.019–0.378 (0.287)	0.313–0.507 (0.371)	0.158–0.419 (0.314)	0.171–0.317
Average measures	0.090–0.752 (0.669)	0.695–0.837 (0746)	0.485–0.783 (0.696)	0.508–0.699
Breathing Raw Scores				
N	39–45	32–40	19–35	11–14
Single measures	0.508–0.680 (0.618)	0.530–0.637 (0.587)	0.655–0.780 (0.681)	0.558–0.664
Average measures	0.838–0.914 (0.890)	0.850–0.898 (0.876)	0.905–0.947 (0.914)	0.863–0.908

*Note*. N = Number of observations per measurement point

### Internal consistency of the modified behavioural BPSN subscale

Cronbach’s Alpha calculated separately for the four GA-groups, are summarized in Table 12. Most Cronbach’s Alpha coefficients were in the range of acceptable to excellent [58] during the heel stick phases of the five measurement points.

**Table 12:** Cronbach’s Alpha for the modified behavioural Bernese Pain Scale for Neonates-subscale, separated by GA-groups

	Cronbach’s Alpha				
	Heel Stick Phase 1 Median (Range)	Heel Stick Phase 2 Median (Range)	Heel Stick Phase 3 Median (Range)	Heel Stick Phase 4 Median (Range)	Heel Stick Phase 5 Median (Range)
Extremely preterm neonates	0.819 (0.813–0.894)	0.821 (0.695–0.862)	0.760 (0.720–0.883)	0.796 (0.690–0.841)	0.830 (0.691–0.840)
*N* = 42–48					
Very preterm neonates	0.908 (0.833–0.915)	0.835 (0.787–0.868)	0.800 (0.705–0.878)	0.794 (0.624–0.902)	0.824 (0.708–0.841)
*N* = 32–44					
Moderate to late preterm neonates	0.836 (0.736–0.932)	0.863 (0.724–0.930)	0.892 (0.844–0.924)	0.872 (0.765–0.896)	0.774 (0.576–0.871)
N = 20–36					
Full-term neonates	0.909 (0.906–0.964)	0.832 (0.813–0.932)			
*N* = 13–20					

*Note*. Median = Median of the coefficients calculated for each rater separately; Range = Range of the five coefficients calculated for each rater

### Correlations between behavioural and physiological indicators of pain

During the heel stick phases of the five measurement points and among the five raters, correlations between the modified behavioural subscale of the BPSN and the item heart rate ranged from *r* = −0.173-0.577 (*Mdn* = 0.196) among extremely preterm neonates, from *r* = 0.024–0.480 (*Mdn* = 0.329) among very preterm neonates, from *r* = − 0.174-0.442 (*Mdn* = 0.172) among moderate to late preterm neonates, and from *r* = − 0.044 to 0.402 (*Mdn* = 0.236) among full-term neonates.

### Concurrent validity

During the heel stick phases of the five measurement points and among the five raters, correlations between the total scale of the modified BPSN and the PIPP-R ranged from *r* = 0.560–0.775 (*Mdn* = 0.683) among extremely preterm neonates, from *r* = 0.582–0.875 (*Mdn* = 0.750) among very preterm neonates, from *r* = 0.603–0.860 (*Mdn* = 0.769) among moderate to late preterm neonates, and from *r* = 0.757–0.898 (*Mdn* = 0.808) among full-term neonates.

### Sensitivity and specificity

The results of the ROC analyses to examine sensitivity and specificity of the modified BPSN total scale separately for each GA-group are provided in Table 13. We found cut-off points needed to increase along with GA to reach about 80% sensitivity and similarly high specificity.

**Table 13:** Results of the ROC analyses for the modified Bernese Pain Scale for Neonates total score, separated for GA-groups

	Heel Stick Phases of Measurement Points t1-t5			
	AUC	Cut-off	Sensitivity	Specificity
	Range (Median)	points	Range (Median)	Range (Median)
Extremely Preterm Neonates	0.707–0.878 (0.801)			
N = 42–48		**0.5**	**0.734–0.875 (0.839)**	**0.398–0.562 (0.538)**
		1.5	0.637–0.765 (0.666)	0.691–0.853 (0.713)
		2.5	0.410–0.594 (0.494)	0.901–0.970 (0.945)
Very Preterm Neonates	0.810–0.930 (0.852)			
*N* = 34–44		0.5	0.849–0.970 (0.905)	0.284–0.606 (0.439)
		**1.5**	**0.745–0.901 (0.811)**	**0.638–0.728 (0.680)**
		2.5	0.596–0.785 (0.648)	0.864–0.977 (0.902)
Moderate to Late Preterm Neonates	0.874–0.941 (0.927)			
*N* = 21–37		1.5	0.900–0.990 (0.970)	0.564–0.660 (0.581)
		**2.5**	**0.763–0.950 (0.897)**	**0.705–0.832 (0.787)**
		3.5	0.532–0.763 (0.675)	0.879–0.975 (0.933)
Full-term Neonates	0.893–0.906			
*N* = 14–20		2.5	0.942–0.959	0.419–0.664
		**3.5**	**0.807–0.888**	**0.808–0.824**
		4.5	0.714–0.831	0.836–0.896
		5.5	0.423–0.751	0.892–0.969

*Note*. The PIPP-R was the reference value, with a cut-off point of 6.5 that discriminated between no/low pain (≤ 6 points) and moderate to high pain (≥ 7 points); AUC = Area under the curve; [95% CI] = 95% confidence intervals of the AUC; the results were originally computed separately for each rater and aggregated assuming normal distribution of the parameter; Range = heel stick phases of measurement points t1-t5; bold-set font = cut-offs with sensitivity and specificity nearest 80%

## Discussion

After rigorous statistical testing, we significantly reduced the number of items in the original BPSN, leaving only three behavioural items: facial expression, crying, and posture. We included only one physiological item, heart rate, in the new version. Psychometric properties of these four items indicate convincing validity across all GA groups, but GA should be considered in pain assessment because different GA-groups require different cut-off points.

### Factor structure and reliability of the BPSN

The factor analysis showed that a model that includes the items crying, facial expression, and posture fits the data best. In fact, facial expression, crying, and body movement are widely studied indicators for pain assessment in neonates and are considered the most sensitive behavioural indicators of pain [4, 59, 60].

Facial expression is considered the most reliable and sensitive indicator for pain assessment in both preterm and full-term neonates [4]. Facial expressions extremely preterm neonates are likely to show include brow bulge, eye squeeze, nasolabial furrow, and vertical mouth stretch [20]. The BPSN more generally assesses facial expression, which aids in assessing preterm infants who wear CPAP masks and tapes to fix tubes to the skin, which can make it difficult to assess specific components of expression, like nasolabial furrow. The PIPP-R item nasolabial furrow was the least frequently rated item in our study, often because it was obscured by CPAP masks or tapes.

Crying is a common pain response in neonates and is included in several pain scales (e.g., [27, 61–63]), but some have questioned crying as an indicator of pain because it cannot be assessed in some neonates [21, 59]. Mechanical ventilation, inhibiting drugs, severe illness, and other reasons may limit the ability to cry. Although crying is not specific to pain [59], it may be the first indication a caregiver has that an infant is in pain [64]. Preterm neonates with immature facial muscles are less able to communicate their pain through facial expressions, so crying can alert their caregivers [17].

Several pain assessment tools include one or more items that assess body movements (e.g., [9, 61, 65, 66]. Holsti, Grunau, Oberlander and Whitfield [67] analysed behavioural pain reaction of early preterm neonates with the Newborn Individualized Development Care and Assessment Program (NIDCAP). They found that neonates flexed and extended their arms and legs, put their hands on their faces, fisted, and finger splayed more often during the heel stick procedure. Morison et al. [68] found neonates with lower GA at birth made more specific body movements but had less facial expression at 32 weeks post-conceptional age, which suggests assessing body movements could provide useful supplementary information about preterm neonates. The BPSN more generally assesses body movement by evaluating a neonate’s posture on a 4-point Likert-scale, ranging from relaxed body to permanent tension. Our results suggest that posture is a sensitive indicator for assessing pain across GA-groups.

We found that heart rate and oxygen saturation did not load on a common physiological factor or correlate with each other. Because heart rate was more sensitive to pain and more strongly associated with the three behavioural indicators of pain, we included heart rate in the new version of the BPSN. The results of our analyses confirm previous findings that correlations between behavioural and physiological indicators of pain were low [69–71], behavioural indicators were more sensitive to pain than physiological indicators [69, 72], and heart rate was more sensitive to pain than oxygen saturation [71].

Though factor loadings of crying, facial expression, and posture did not vary within raters during the heel stick phases, they did vary between raters. This result suggests that different raters assess pain differently, an assumption further supported by the results of our interrater reliability analysis. There was good to excellent interrater agreement on crying, but agreement on facial expression and posture ranged from poor to good [73], depending on the measurement point and the model to calculate ICCs. The differences in interrater reliability could be explained by differences in the way raters defined the items. Crying may be a more objective and reliable item than facial expression or posture because it considers duration. Improving the guidelines and training for applying the BPSN may improve interrater agreement.

The first validation study of the BPSN [24] used Cronbach’s Alpha reliability coefficient to calculate interrater reliability, and found interrater reliability of the subjective subscale of the BPSN (*r* = 0.77–0.97) was high. Cronbach’s Alpha determines if the ratings of two or more persons are consistent, but it does not measure absolute agreement [74]. Since the cut-off differentiates between a painful and non-painful state, agreement between nurses and other caregivers about an infant’s level of pain is crucial. We thus decided to use the more stringent absolute agreement model to calculate interrater reliability.

Interrater agreement and factor loadings of the items crying, facial expression, consolation, and posture tended to decrease over time. Cronbach’s Alpha and corrected item-total correlations of the items crying, facial expression, and posture tended to decrease too. This accords with the results of another study that showed high within-subject variability among preterm neonates’ pain reaction across repeated measurement points [75]. Interrater reliability was high during the heel sticks 1–3 and decreased during heel sticks 4–5. These findings cannot be explained by rater fatigue, because the video sequences were analysed in random order. The variability in pain reactions might be explained by the influence of individual contextual factors and needs to be investigated [1, 2, 20, 21].

### Validity of the modified BPSN

The modified BPSN that includes crying, facial expression, posture, and heart rate showed good construct validity and concurrent validity with the PIPP-R. Pain scores on the behavioural subscale averaged more than one point higher during the heel stick than during the baseline and recovery phases. Pain scores on heart rate averaged 0.65 points higher during the heel stick phase than during the recovery phase. Neonates’ GA at time of birth influenced their pain scores. With every additional week of GA, pain scores on the behavioural subscale (crying, facial expression, posture) increased about 0.063 points. If we apply this result on our study sample with a wide range of GAs (24 2/7–42 5/7 weeks of GA), behavioural pain reaction of the neonate with the highest GA was about 1.13 points higher than pain reaction of the neonate with the lowest GA. Heart rate of the neonate with the highest GA was also about 0.76 points higher than heart rate of the neonate with the lowest GA. Like other studies that analysed the relationship between gender and pain reaction in neonates (e.g., [76–78]), we found gender had no effect on the level of pain scores.

### Sensitivity and specificity of the modified BPSN

The results of the sensitivity and specificity analyses suggest that a cut-off of 1.5 points (total overall score = 12 points) would discriminate between no to low pain and moderate to high pain (measured with the PIPP-R). For the original BPSN scale, the cut-off was much higher, at 10.5 points (total overall score = 27 points). We found that the mean of the BPSN total scale that included nine items varied widely and depended on the rater, but it did not reach the cut-off value of 11 points during the heel stick phases of the five measurement points. The preliminary dose of oral sucrose administered to neonates before each heel stick may have lowered pain scores in our study [28]. In the first validation study of the BPSN, neonates received no pain relieving intervention before the heel stick, and BPSN total scores increased significantly during the heel stick, averaging 15.96 points (*SD* = 5.7) [24]. The relief provided by sucrose should be factored into the decision about a new cut-off value for the modified BPSN.

### Comparison of different GA-groups

Neonates with younger GA at birth had lower pain scores than more mature infants. The results of the separate sensitivity and specificity analyses for the four GA-groups indicated as GA increases, so should the cut-off of the BPSN that discriminates between no to low pain and moderate to high pain (measured with the PIPP-R). To reach a sensitivity and specificity of approximately 80%, extremely preterm neonates require a cut-off value of 0.5 points, very preterm neonates require 1.5 points, moderate to late preterm neonates require 2.5 points, and full-term neonates require 3.5 points. Our ROC analysis showed that the modified BPSN was least able, but still moderately good [41], to discriminate between neonates who experience no or low pain and neonates who experience moderate to high pain in the group of extremely preterm neonates and increases with increasing GA. Extremely preterm neonates’ pain expression may be less apparent because their immature nervous system and facial muscles prevent them from expressing a robust pain reaction [20, 21, 60, 68]. Understanding the difficulty this poses for accurate pain assessment in extremely preterm neonates could be helpful when establishing cut-off values for the BPSN. Based on our study results, we recommend differentiating between GA-groups and establishing cut-off values based on GA. The PIPP-R already includes GA in pain assessment; the younger the GA, the more points PIPP-R adds to the pain score [26].

The other analyses we conducted separately for the four GA-groups showed that concurrent validity of the modified BPSN total score with the PIPP-R was highest for full-term neonates (*r* = 0.814–0.834) and lowest, but still good, for extremely preterm neonates (*r* = 0.631–0.710). Interrater agreement on facial expression and posture tended to improve as GA increased.

### Limitations

This study is limited, first, by our decision to rate neonates’ pain expression from video sequences. Characteristics of the videos may have affected the reliability of the ratings (e.g., poor lighting conditions, quality of the raters’ screen, position of the neonate, several assistants for video recording). Second, different nurses performed the heel sticks, and their individual characteristics may have influenced neonates’ pain reaction. Third, particularly during the baseline and recovery phases, where the scores of the items were low, floor effects may have influenced our study results. For example, we considered a variety of extensions of the model specification in our factor analysis but discarded them because of convergence problems likely related to floor effects, when upper categories were almost or completely left empty. Treating the rating scores as numeric did not resolve floor effect problems, or rather the opposite [79], but allowed to obtain results. Floor effects may also have lowered interrater agreement, especially during the baseline and recovery phases. Fourth, our later hypothesis testing may be compromised by measurement error caused by low interrater agreement [40]. We compensated for this possible problem by either including the raters in the model, or by conducting separate analyses for each rater and then pooling the results. Fifth, pain reaction was measured during the heel stick, so our results cannot be generalized to other acute painful procedures or more persistent or chronic pain. The BPSN is used for routine pain assessment in NICUs and should therefore be sensitive to repeated and more prolonged and chronic pain, so future validation studies should assess and compare the level of pain scores during different painful situations.

## Conclusions

The modified version of the BPSN that includes facial expression, crying, posture, and heart rate is a promising tool for assessing acute pain in full-term and preterm neonates across gestational ages, but our results suggest that adding different cut-off points for different GA-groups will improve the BPSN’s clinical usefulness.

## Abbreviations

AUC: Area under the curve
BPSN: Bernese Pain Scale for Neonates
CFA: Confirmatory factor analysis
CFI: Comparative Fit Indices
CPAP: Continuous positive airway pressure
GA: Gestational age
ICC: Intraclass Correlation Coefficient
*M*: Mean
*Mdn*: Median
NICU: Neonatal intensive care unit
NIDCAP: Newborn Individualized Development Care and Assessment Program
OLInv: Overall loadings invariance
PIPP-R: Premature Infant Pain Profile-Revised
RMSEA: Root mean square errors of approximation
ROC: Receiver-operating characteristic
*SD*: standard deviation
TLI: Tucker-Lewis Indices
WRLInv: Within-rater loadings invariance
